# Production of reactive oxygen species and involvement of bioprotectants during anhydrobiosis in the tardigrade *Paramacrobiotus spatialis*

**DOI:** 10.1038/s41598-022-05734-6

**Published:** 2022-02-04

**Authors:** Ilaria Giovannini, Thomas C. Boothby, Michele Cesari, Bob Goldstein, Roberto Guidetti, Lorena Rebecchi

**Affiliations:** 1 Department of Life Sciences, University of Modena and Reggio Emilia, Via G. Campi 213/D, 41125 Modena, Italy; 2 Department of Molecular Biology, University of Wyoming, Laramie, WY USA; 3 Department of Biology, University of North Carolina at Chapel Hill, Chapel Hill, NC USA

**Keywords:** Evolution, Zoology

## Abstract

Water unavailability is an abiotic stress causing unfavourable conditions for life. Nevertheless, some animals evolved anhydrobiosis, a strategy allowing for the reversible organism dehydration and suspension of metabolism as a direct response to habitat desiccation. Anhydrobiotic animals undergo biochemical changes synthesizing bioprotectants to help combat desiccation stresses. One stress is the generation of reactive oxygen species (ROS). In this study, the eutardigrade *Paramacrobiotus spatialis* was used to investigate the occurrence of ROS associated with the desiccation process. We observed that the production of ROS significantly increases as a function of time spent in anhydrobiosis and represents a direct demonstration of oxidative stress in tardigrades. The degree of involvement of bioprotectants, including those combating ROS, in the *P. spatialis* was evaluated by perturbing their gene functions using RNA interference and assessing the successful recovery of animals after desiccation/rehydration. Targeting the glutathione peroxidase gene compromised survival during drying and rehydration, providing evidence for the role of the gene in desiccation tolerance. Targeting genes encoding glutathione reductase and catalase indicated that these molecules play roles during rehydration. Our study also confirms the involvement of aquaporins 3 and 10 during rehydration. Therefore, desiccation tolerance depends on the synergistic action of many different molecules working together.

## Introduction

Insufficient hydration is extremely stressful for living organisms as water is required for metabolic reactions. Nevertheless, some animals living in habitats subject to periodic or unpredictable desiccation periods have evolved a remarkable adaptive strategy called anhydrobiosis. This physiological state allows for the nearly complete dehydration of the animal’s body and cells, a reduced or suspended metabolism leading to a temporary suspension of active life processes, and finally a developmental standstill as a direct response to the desiccation of the surrounding habitat of the animals^1–4^. This state is reversed by the reappearance of environmental water, with the restoring of metabolism and of all features of active life, even after the permanence in a desiccated state for months to decades^1,2,4–6^. However, anhydrobiosis, as well as the time spent in a desiccated state, can still cause cellular damages, including membrane destabilization, protein and nucleic acid denaturation, metabolic dysregulation, and oxidative stress with the eventual death of organisms^7–16^.

To withstand stress caused by the drastic loss of body water, many anhydrobiotic animals first slow the rate of their dehydration by modifying their body in a compact structure that reduces the surface area, and consequently the evaporation rate^17–19^. Simultaneously, they undergo biochemical changes associated with the synthesis of molecules working as bioprotectants, which replace cellular water or stabilize cellular machinery^13,20–27^. To gain insights into anhydrobiosis and what bioprotectants help to mediate this phenomenon, tardigrades are an attractive model.

Tardigrades are hygrophilous animals that need to be surrounded by a film of water to perform active life. As a consequence, their capability to colonise terrestrial environments (e.g. moss, lichen, soil, and leaf litter) is linked to their well-known ability to enter anhydrobiosis at any stage of their life cycle, from egg to adult^28^. As their surroundings lose water, tardigrades lose most (> 95%) of their free and bound body water and suspend their metabolism^3^. The loss of water is accompanied by the invagination of the intersegmental cuticle and by the retraction of the head and legs, resulting in a compact tun-shaped body^3,29,30^. Anhydrobiotic (desiccated) tardigrades can withstand several physical and chemical extremes (e.g. very low and high temperature, high pressure, vacuum, organic solvents and radiations) that are far more extreme than that imposed by their natural habitats, including space conditions^3,25,26,31–36^. As well as in other anhydrobiotic animals, the desiccation in tardigrades is accomplished through morphological changes (tun) together with the synthesis of different bioprotectants^20–22,24–27^. These molecules include, for example, osmolytes, trehalose, antioxidants, Heat Shock Proteins (HSPs), Aquaporin Proteins (AQPs) and Intrinsically Disordered Proteins (IDPs), including the Late Embryogenesis Abundant Proteins (LEA)^13,20–22,24–27^. Trehalose, a disaccharide with high ability for water-replacement during dehydration, immobilising macromolecules and stabilizing cellular structure through vitrification has long been implicated in desiccation tolerance^20,26,37^. The presence and the amount of trehalose detected in tardigrades are different from species to species^27^, but is much lower than the one recorded in other anhydrobiotic animals that use trehalose as mechanism to reduce damages induced by desiccation^1,38–40^. Therefore, the degree of involvement of trehalose as a protective molecule in anhydrobiotic tardigrades is still debated^27^. Antioxidants, such as scavenging enzymes, limit oxidative stress, which appears to be one of the most deleterious damages associated with desiccation^9^. The depletion of water changes the ionic concentration of anhydrobiotic organisms, including tardigrades^41^, leading to the formation of reactive oxygen species (ROS)^9,11,42,43^, consisting in free radicals [e.g. superoxide anion radical (O_2_^−^), hydroxyl radical (OH·), perhydroxy radical (HO_2_·)] and non-radical forms [e.g*.* hydrogen peroxide (H_2_O_2_) and singlet oxygen (^1^O_2_)]^44^. Water loss therefore increases the susceptibility of biomolecules to the attack of reactive oxygen molecules, damaging proteins, membranes, and DNA^9,11,13,22,27,42,43^. Since aquaporins contribute to water transport through plasma membranes, some researchers have suggested that they play some role in anhydrobiosis, hypothesizing their involvement in the regulation of water loss to fine tune desiccation kinetics^11,23,45–47^. However, it remains unclear if aquaporins function in preparing anhydrobiotic animals for desiccation or play a role in rehydration.

**Figure 1:**
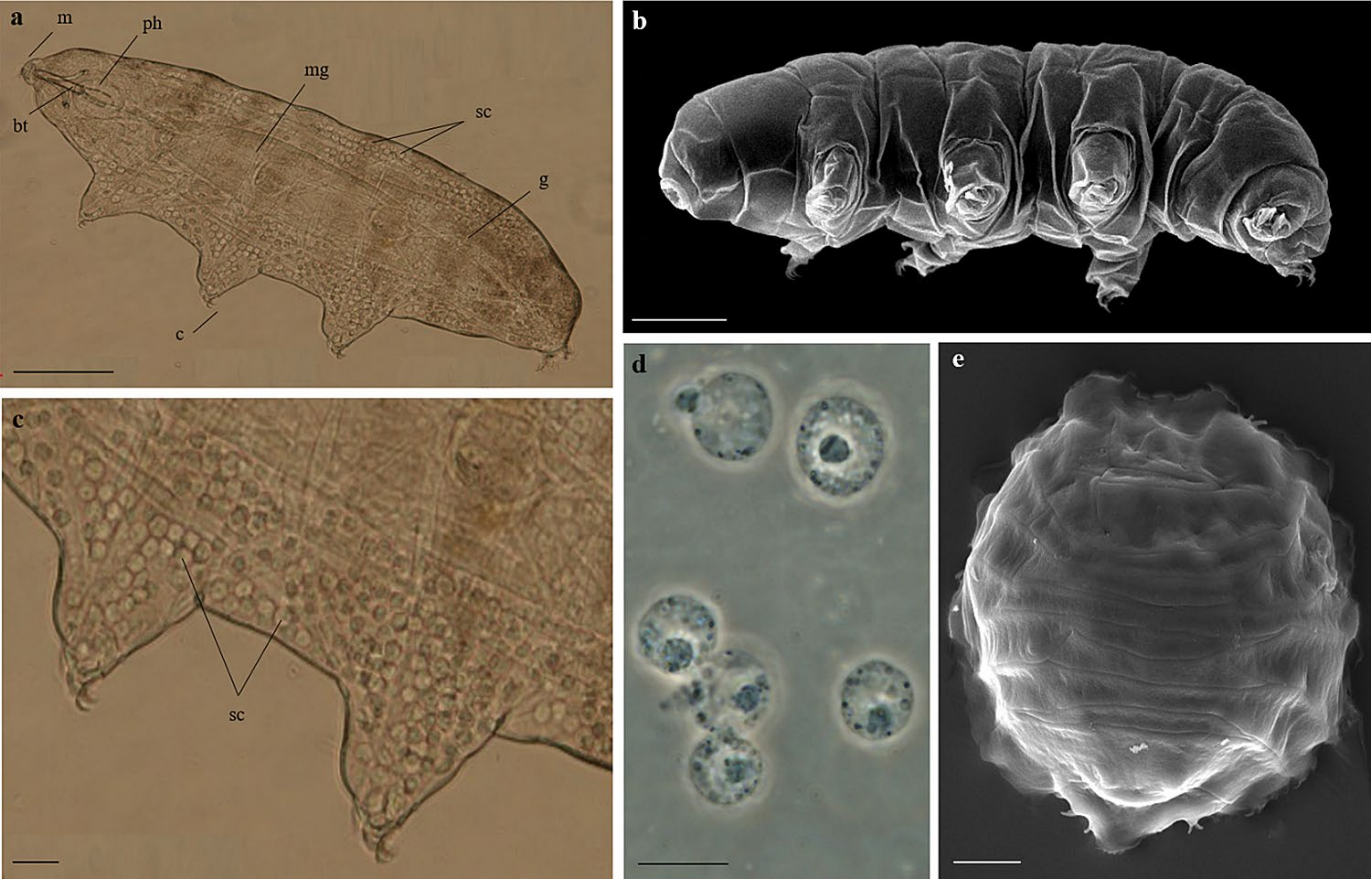
*Paramacrobiotus spatialis*. (**a**) Specimen in toto and in vivo. (**b**) Specimen in toto. (**c**) Magnification of image (**a**) showing storage cells in the body cavity in correspondence to the second and third pair of legs. (**d**) Storage cells in vivo. (**e**) Desiccated specimen (tun). (**a**,**c**,**d**) LM (PhC), (**b**,**e**) SEM. Scale bars: a = 100 µm, b = 50 µm, c, e = 20 µm, d = 10 µm. bt = buccal tube; c = claws; m = mouth; mg = midgut; g = gonad; ph = pharynx; sc = storage cells.

In this paper, the eutardigrade *Paramacrobiotus spatialis* Guidetti et al., 2019 was selected as a model organism (Fig. 1) in light of its robust ability to survive desiccation^10,48^. To understand what bioprotectants might be involved in mitigating the stresses imposed by desiccation, we first sought to identify exactly what those stresses are. As mentioned above, water loss can lead to the generation of deleterious ROS, however this has never been directly tested before in tardigrades. To verify whether desiccation induces production of ROS in tardigrades, we analysed the ROS production in the coelomocytes (storage cells; Fig. 1c). Coelomocytes are round free-floating cells in the body cavity of tardigrades which act as reservoirs and transporters of lipids and glycogen^49–52^. The number of storage cells in a tardigrade specimen is in the range of a few hundred up to more than 1000 cells according to the species^4,52,53^, while their size changes along with animal life cycle including oocyte developmental in females^54^. Contrasting data are reported about the decline in size and number of storage cells after a period of anhydrobiosis in tardigrade species^51–53^. A decline in size of storage cells is due to the use of stored material as energy requirements to enter and exit anhydrobiosis^51^.

Next, we investigated possible roles for endogenous protectants, such as antioxidant enzymes, aquaporin proteins and the sugar trehalose, in desiccation tolerance. Genes encoding five antioxidant enzymes (catalase, superoxide dismutase, glutathione peroxidase, glutathione transferase and glutathione reductase), for two aquaporins (aquaporins 3 and 10) and for the enzyme trehalose-6-phosphate synthase were thus targeted using RNA interference (RNAi), and later the successful recover of *P. spatialis* specimens after desiccation was verified.

## Results

### Production of reactive oxygen species (ROS) in anhydrobiosis

**Figure 2:**
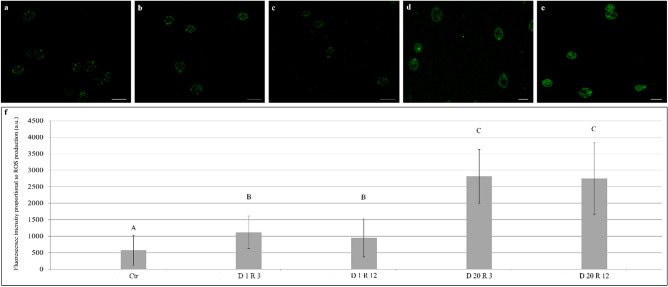
Intensity of fluorescence signal emitted by storage cells of the tardigrade *Paramacrobiotus spatialis* as a marker of ROS production, detected 3 h and 12 h after the rehydration process (R) in animals kept in a desiccated state (D) for 1 day and 20 days, using the fluorescent probe 2,7-dichlorodihydrofluorescein diacetate (DCFH_2_-DA). (**a**) Hydrated animals (control). (**b**) Animals kept in a desiccated state for 1 day (3 h after rehydration process). (**c**) Animals kept in a desiccated state for 1 day (12 h after rehydration process). (**d**) Animals kept in a desiccated state for 20 days (3 h after rehydration process). (**e**) Animals kept in a desiccated state for 20 days (12 h after rehydration process). (**a**–**e**) Scale bars = 10 µm. (**f**) Each column represents the mean value of the fluorescence intensity emitted by the storage cells for each experimental group. The total number (n) of measured cells is: Ctr, n = 138; D 1 R 3, n = 46; D 1 R 12, n = 78; D 20 R 3, n = 78; D 20 R 12, n = 36 (see (see Table S1). The bar on each column represents standard deviation. Different letters above each column indicate significant differences in the fluorescence signals among the different experimental conditions. a.u. = arbitrary unit. D 1 = animals kept in a desiccated state for 1 day, D 20 = animals kept in a desiccated state for 20 days, R 3 = 3 h after the rehydration process, R 12 = 12 h after the rehydration process, Ctr = control animals. The storage cells of desiccated animals always emitted an intense fluorescence signal, while those of control ones emitted a faint spotted fluorescence signal (Fig. 2). The intensity of the fluorescence signal emitted by the storage cells of desiccated animals was always significantly higher than the fluorescence intensity detected in cells of control animals (Fig. 2; Table 1; one-way ANOVA: n = 485; *p* < 0.001). Moreover, the intensity of the fluorescence signal was at its highest level in the coelomocytes of animals kept desiccated for 20 days, while the lowest signal was recorded in cells of tardigrades kept desiccated for 1 day (Fig. 2; Table 1).

The overall level of intracellular Reactive Oxygen Species (ROS) was assessed using the fluorescent probe 2,7-dichlorodihydrofluorescein diacetate (DCFH_2_-DA)^55,56^. The ROS production in anhydrobiosis was determined measuring the intensity of the fluorescent signal emitted by storage cells of the desiccation tolerant tardigrade *P. spatialis*. Storage cells are particularly useful as they are numerous as well as the only cell type within the tardigrade body cavity. Storage cells are also easy to obtain and manipulate, whereas other tissues in tardigrades are more difficult to obtain and work with (Fig. 1c,d). Figure 2 shows the fluorescence signals detected respectively 3 h and 12 h after the rehydration process in tardigrade storage cells previously kept in a desiccated state for 1 day and 20 days, and in the animals kept in a hydrated state as control.

**Table 1:** Statistical comparisons (one-way ANOVA, Tukey post-hoc test) among fluorescence signals emitted by the storage cells of *Paramacrobiotus spatialis* at different experimental conditions.

	Control	D 1 R 3	D 1 R 12	D 20 R 3	D 20 R 12
Control		*p* < 0.01	*p* < 0.01	*p* < 0.001	*p* < 0.001
D 1 R 3	*p* < 0.01		n. s.	*p* < 0.001	*p* < 0.001
D 1 R 12	*p* < 0.01	n. s.		*p* < 0.001	*p* < 0.001
D 20 R 3	*p* < 0.001	*p* < 0.001	*p* < 0.001		n. s.

The total number (n) of measured cells is: Control, n = 138; D 1 R 3, n = 46; D 1 R 12, n = 78; D 20 R 3, n = 78; D 20 R 12, n = 36 (see Table S1).

D 1 = animals kept in a desiccated state for 1 day, D 20 = animals kept in a desiccated state for 20 days, R 3 = 3 h after the rehydration process, R 12 = 12 h after the rehydration process, n.s. = not significant.

### Disruption of target genes function

RNA interference (RNAi) was used to disrupt the function of genes encoding proteins potentially involved in desiccation tolerance in the tardigrade *Paramacrobiotus spatialis.* RNAi has previously been characterized in tardigrades and is known to target specific gene products for destruction both in adults during desiccation and in embryos during development^24,57^. Similarly, it has been shown that large amounts of non-specific dsRNA (e.g*.* double stranded RNA targeting GFP) do not affect tardigrade survival^24^ nor embryo viability and development^57^. In this study, the eight genes targeted by RNAi were: *gpx*, *gr*, *gst*, *cat* and *sod*, encoding for enzymes (glutathione peroxidase, glutathione reductase, glutathione transferase, catalase, and superoxide dismutase, respectively) that counteract reactive oxygen species; *tps*, encoding for the enzyme trehalose-6-phosphate synthase, involved in the pathways of trehalose production; *aqp 3* and *aqp 10*, encoding for aquaporin proteins 3 and 10, which are presumably involved in the rapid transport of water through cell membranes.

The involvement of these target genes was verified by comparing the motilities of *P. spatialis* animals that have exited tun belonging to three groups: 1. uninjected control animals (control 1); 2. injected with RNase free water control animals (control 2); 3. injected animals with the double stranded RNA (dsRNA) of the target gene (experimental group). The motility of the animals after a period of anhydrobiosis was evaluated by monitoring locomotion performance (i.e. coordinate movements of the body) immediately after the rehydration process (t_0_), 1 h (t_1_), 24 h (t_24_), and 48 h (t_48_) later.

Additionally, five specimens of *P. spatialis* were individually injected with dsRNA of each target gene, desiccated, and then rehydrated. A reverse transcriptase-polymerase chain reaction (RT-PCR) showed a marked decrease in the expression level of each target gene in comparison to DNA polymerase II (*DNA pol II*) used as a control gene (Fig. S1), indicating that RNA interference led to a decrease in the targeted gene’s expression level.

**Figure 3:**
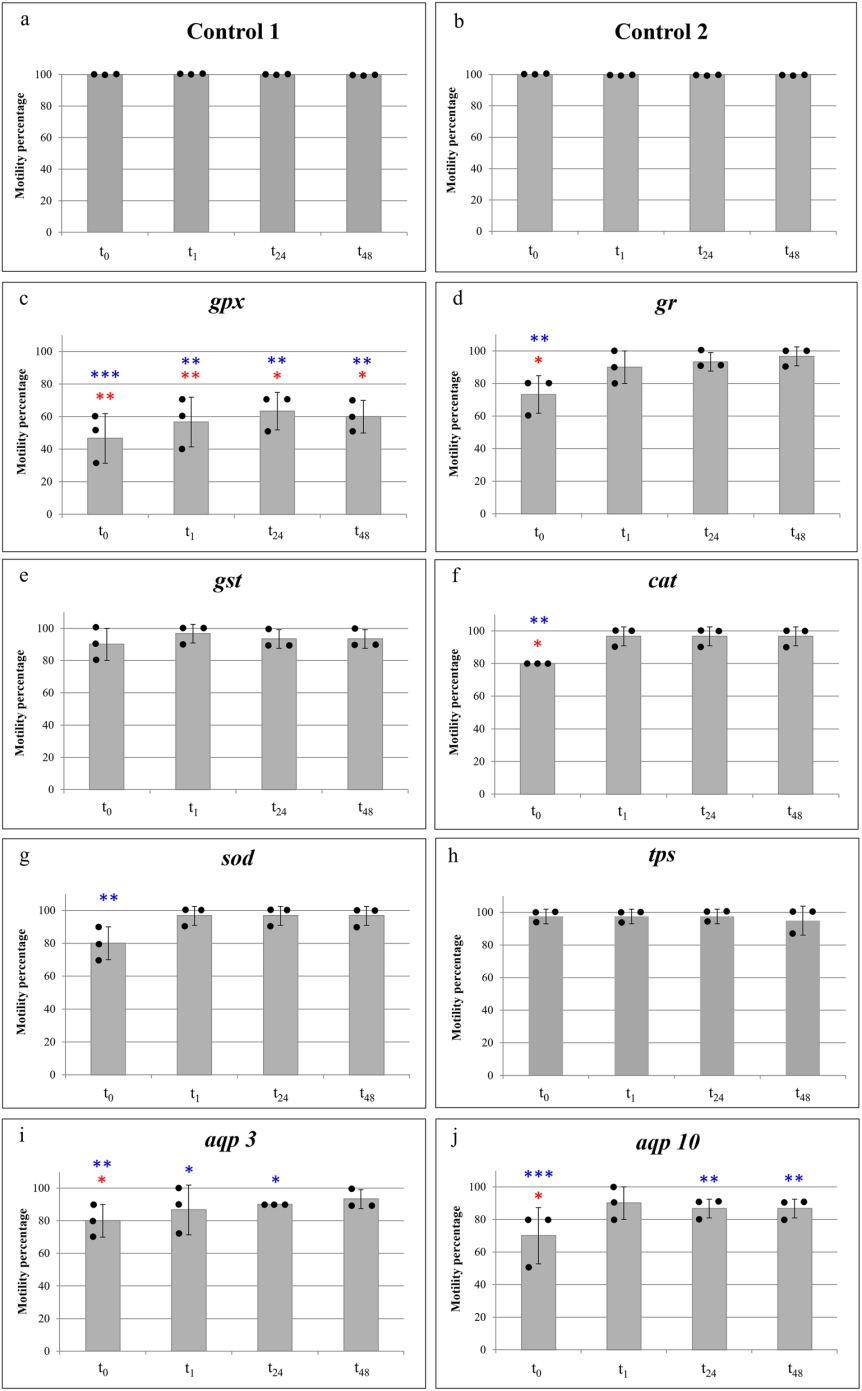
Percentages of tardigrades with motility recorded immediately after the rehydration process (t_0_), 1 h (t_1_), 24 h (t_24_) and 48 h (t_48_) later in control animals and in specimens of *Paramacrobiotus spatialis* injected with dsRNA of the target genes. (**a**) Control 1: uninjected animals. (**b**) Control 2: animals injected with RNase free water. Animals injected with dsRNA of: (**c**) *gpx* gene encoding glutathione peroxidase enzyme; (**d**) *gr* gene encoding glutathione reductase enzyme; **(e)** *gst* gene encoding glutathione transferase enzyme; (**f**) *cat* gene encoding catalase enzyme; (**g**) *sod* gene encoding superoxide dismutase enzyme; (**h**) *tps* gene encoding trehalose-6-phosphate synthase enzyme; (**i**) *aqp 3* gene encoding aquaporin 3; (**j**) *aqp 10* gene encoding aquaporin 10. Each column represents the mean value of three replicates and the bar on each column represents the standard deviation. The black dots on each column represent the values of each replicate composed of 10 specimens each. Blue asterisks above columns show significant statistical differences (Kruskal–Wallis test) with respect to control 1 and 2. Red asterisks above columns show significant statistical differences (Kruskal–Wallis test) with respect to *tps* gene, used as a further control (Control 3) to exclude the possibility that the injection of large amount of dsRNA is harmful for *P. spatialis.*

The assessment of motility in both control 1 (uninjected) and control 2 (water injected) animals always resulted 100% (Fig. 3a,b), since all animals are alive at each time point assayed after the rehydration process. These data demonstrate that the desiccation protocol was suitable for *P. spatialis*, and that the injection alone does not affect survival of the tardigrades during anhydrobiosis. On the other hand, significant differences were recorded between the motility percentages of both controls and most experimental groups (Fig. 3; Kruskal–Wallis test: number of pairwise comparisons = 132; *p* < 0.001). For treatments targeting antioxidant enzymes, specimens injected with dsRNA of *gpx* showed significantly lower motilities with respect to both types of controls at every time after the rehydration process (t_0_: *p* < 0.001; t_1_: *p* < 0.01; t_24_: *p* < 0.01; t_48_: *p* < 0.01; Fig. 3c). In animals injected with dsRNA for genes of *gr*, *cat* and *sod*, significant differences with respect to both types of controls were only observed between the percentages of animal motilities recorded at t_0_ (immediately after the rehydration process) (*gr*: *p* < 0.01; *cat*: *p* < 0.01; *sod*: *p* < 0.01; Fig. 3d,f,g). Significant differences in the motility percentages with respect to both types of controls were also evidenced at t_0_, t_1_ and t_24_ in animals injected with dsRNA of *aqp 3* (t_0_: *p* < 0.01, t_1_: *p* < 0.05; t_24_: *p* < 0.05; Fig. 3i) and at t_0_, t_1_ and t_24_ in animals injected with dsRNA of *aqp 10* (t_0_: *p* < 0.001, t_24_: *p* < 0.01; t_48_: *p* < 0.01; Fig. 3j).The motility percentages of animals injected with dsRNA of the *gst* and *tps* genes did not show significant differences with respect to both types of controls (uninjected and water injected) at every time after the rehydration process (Fig. 3e,h). Since RNAi knockdown of the *tps* gene resulted in negligible effects on the animal motilities at all times following rehydration, and since the *tps* dsRNA was the largest used in our study (Table S2), we consider this gene as a further control (control 3), and further evidences that the injection of large amount of dsRNA is not harmful for *P. spatialis*. The motility percentages of animals injected with dsRNA targeting the *tps* gene compared with the motility percentages of animals injected with dsRNA of other target genes shows significant differences (Table S3). These statistical differences are consistent with the scored significances of comparisons between controls (non-injected and water injected) and target genes, except for motility at t_0_ of animals injected with *sod* dsRNA, motilities at t_1_ and t_24_ of animals targeted for *aqp 3* and motilities at t_24_ and t_48_ of animals targeted for *aqp 10* (Fig. 3; Table S3). Therefore, only for these exceptions is it possible to presume an effect on *P. spatialis* motilities was due to injection of large dsRNA.

## Discussion

This study provides an evaluation of oxidative stress production during desiccation in tardigrades through the direct assessment of the production of reactive oxygen species (ROS) in storage cells. In addition, this study assesses the degree of involvement of various bioprotectants in anhydrobiotic survival of tardigrades using RNA interference to perturb gene functions in *Paramacrobiotus spatialis.*

Storage cells are singular free-floating cells accumulating polysaccharides, lipids, and proteins^49,50,58^. The faint spotted fluorescence emitted by the storage cells of the control specimens of *P. spatialis* denotes that in metabolically active animals there is a regular, but small, production of ROS due to the standard metabolic reactions. This has been reported in all living organisms, not only related to anhydrobiosis (see França et al.^9^; Kranner & Birtić^43^; Boveris & Chance^59^). The storage cells of desiccated specimens of *P. spatialis* emitted an intense fluorescence, demonstrating that anhydrobiosis causes a significant increase in ROS production and consequently of oxidative stress.

Previous reports evaluated the oxidative stress associated with desiccation in tardigrades indirectly, by assessing the antioxidant defences mounted in response to drying in whole animals^22,46^, or by evaluating the ROS-damaged proteins that can accumulate as carbonylated products during the anhydrobiotic period^60^. Evidence of the production of free radicals (and consequently of oxidative stress) in anhydrobiosis has been found in other organisms, such as cyanobacteria (see Potts^61^), the yeast *Saccharomyces cerevisiae* (see Pereira et al.^62^)*,* intertidal seaweeds (see Flores-Molina et al.^63^), the moss *Fontinalis antipyretica* (see Cruz de Carvalho et al.^64^) and the shrimp *Marsupenaeus japonicas* (see Duan et al.^65^).

In tardigrades, ROS production occurs both during desiccation, and while in a desiccated state. These data can explain the increase in mortality and/or in a long recovery time (i.e. time to recover active life after desiccation) measured in *P. spatialis* specimens after extended periods in a desiccated state^10^. These two phenomena are directly proportional to the time spent in a desiccated state, since molecular damages are accumulated with time because metabolism is arrested, and repair systems are not working^10^. Oxidative stress leads to a long recovery time and to the death of tardigrades since the animals need more time to repair oxidation damages.

The glutathione pathway, including the enzymes glutathione peroxidase, glutathione transferase and glutathione reductase, is an important antioxidant system involved in the scavenging of ROS^13^. In this pathway, the glutathione peroxidase and glutathione transferase act by removing hydrogen peroxide, while the glutathione reductase reduces the glutathione disulphide to glutathione^9,43^. Targeting a gene encoding the antioxidant enzyme glutathione peroxidase, which is induced by desiccation, compromised survival during desiccation, providing evidence for this gene’s role in tardigrade desiccation tolerance. Targeting of *gpx* by RNAi in the tardigrade *P. spatialis* resulted in a significant lower percentage of tardigrades with motility (i.e. lower survival) at every time after the rehydration process with respect to control animals kept hydrated and metabolically active (Controls 1 and 2) and with respect to animals targeted for *tps* (non-effective gene, Control 3). The effects on the percent motility observed in animals injected with dsRNA of *gpx* are enhanced by the presence of low copies of this gene in tardigrades^25^. A similar pattern was obtained with the knockdown of glutathione peroxidase transcripts by RNAi in the nematode *Panagrolaimus superbus*, which led to a decrease survival post-desiccation^66^. The glutathione peroxidase enzyme has a crucial role during anhydrobiosis of *P. spatialis*, since animals targeted for *gpx* do not recover their motility indicating that other antioxidant enzymes or other endogenous molecules cannot fully replace essential roles of glutathione peroxidase. The important role of this molecule in desiccation tolerance of *P. spatialis* extend the previous results of Rizzo et al.^22^, where glutathione peroxidase was one of the enzymes with the higher enzymatic activity. This enzyme is utilised to counteract ROS not only during the dehydration process, but also probably plays a vital role during the rehydration phase^22^ and thus the enzyme encoded by *gpx* is essential for tardigrade anhydrobiosis. The crucial role of glutathione peroxidase in tardigrades is in line with its role in counteracting oxidative stress caused by desiccation in other organisms: e.g. in transgenic tobacco plants^67^, in the larvae of the midge *P. vanderplanki*^11^, in the dauer larvae of the nematode *Caenorhabditis elegans*^68^, and other species of nematodes^66,69,70^.

The targeting of glutathione transferase (*gst*) had no effects on the motilities of injected animals at any time following rehydration. GSTs have been seen to exist in high copy number in several tardigrade species^25,71^, implying that *P. spatialis* may also have a high copy number of these genes. If this is the case, the knockdown of a single putative gene of *gst* might not be sufficient to perturb anhydrobiosis in this species.

The data obtained on *P. spatialis* specimens targeted for glutathione reductase (*gr*) gene indicate that this molecule plays a role in the course of rehydration. Indeed, tardigrades targeted for this gene are immobile immediately after the rehydration process (t_0_), but then they recover their motility (t_1_–t_48_). The *gr* might eliminate the ROS accumulated during desiccation, but when it is targeted the ROS production is not completely counteracted and the targeted animals need more time to recover motility. Therefore, it is possible to presume that other endogenous molecules not targeted in these specimens take on the activities made by the *gr* during rehydration, and therefore allow animals to recover their motilities.

Another defence counteracting the ROS toxicity involves the system made up by superoxide dismutase and catalase. The first destroys free radical superoxide converting it in H_2_O_2_ which is then reduced by catalase^72^. The data obtained on specimens of *P. spatialis* whose for catalase (*cat*) gene had been perturbated by RNAi indicate that this molecule plays a role in the course of rehydration. Indeed, tardigrades targeted for this gene are immobile immediately after the rehydration process (t_0_), but then they recover their motility (t_1_–t_48_). Since the action of catalase is similar with that of glutathione peroxidase, it is probable that in animals targeted for catalase, the glutathione peroxidase takes on its function during rehydration. In other species of tardigrades and in other desiccation tolerant animals, experiments confirm the role of this enzyme in the desiccation process to prevent oxidative stress^11,47,68,73,74^. An upregulation of catalase gene during anhydrobiosis was detected in the tardigrade *H*. *exemplaris* (see Yoshida et al.^47^), in the midges *Belgica antarctica* and *P. vanderplanki* (see Cornette & Kikawada^11^; Lopez-Martines et al.^73^) and in the nematodes *Aphelenchoides fragariae* and *C. elegans* (see Erkut et al.^68^; Fu et al.^74^). Targeting superoxide dismutase (*sod*) via RNAi did not negatively affect the motility percentages of injected animals at any time of rehydration. Comparing the gene repertoire of different tardigrade species, the duplication of *sod* genes was observed as a common characteristic of anhydrobiotic tardigrades^25,71^. Since *P. spatialis* is a good anhydrobiont^10,48^, it is possible that its *sod* genes are duplicated as well, and therefore, the targeting of a single *sod* gene might not be enough to affect the motilities of injected tardigrades, even though the *sod* genes could play a useful role during desiccation.

The trehalose-6-phosphate synthase (TPS) enzyme is involved, together with the enzyme trehalose-6-phosphate phosphatase, in the biosynthesis pathway of the sugar trehalose. This sugar acts as a common water replacement molecules and stabilizer of biological structures by protecting cellular systems from dehydration and preventing protein aggregation and eliminating ROS^1,27,37,75^. Despite most anhydrobiotic metazoans, such as the midge *P. vanderplanki* and the cysts of *Artemia* spp., use trehalose as a protective mechanism^1,38–40^, not all tardigrade species produce this sugar, and when they produce it, the amount of trehalose differs among species and it is always very low in comparison with other anhydrobiotic animals^20,39,76^. The detection of trehalose in *Paramacrobiotus* species^20^ and of the *tps* gene in *P. spatialis*^77^, allowed us to target this gene in this study, demonstrating that its targeting did not significantly affect animal motility after a cycle of desiccation/rehydration. In line with this, a recent study showed the presence of three TPS-TPP genes in *Paramacrobiotus* sp.^78^, and probably the targeting of a single copy of *tps* gene cannot be sufficient to have an effect on the motilities of injected animals. Our results demonstrate that trehalose is not the only molecule involved in desiccation tolerance, in line with the concept suggested by Hibshman et al.^27^ that in desiccation tolerance of some tardigrades other molecules must have protective effects that substitute for the function of trehalose. Therefore, trehalose represents only one possible evolutionary pathway towards desiccation tolerance, and probably, is not the most crucial biochemical adaptation to protect cell during desiccation in tardigrades. This agrees with other completely desiccation tolerant organisms, namely bdelloid rotifers, that are unable to produce trehalose^79^. Rotifers protect the cells under desiccation stress utilizing different types of molecules, such as LEA proteins, hydrophilins, chaperones (i.e. Heat Shock proteins), antioxidants, and molecules involved in DNA repair^6^.

Aquaporins are integral membrane proteins present in all domains of life, ranging from archaea and bacteria to eukaryotes^80^. They play an intrinsic role in the physiological movements of water across cell membranes, and consequently through the entire organism^11,81^. They could be an adaptive response to changing water abundance and osmolality in the organism’s habitat^82^, and to water loss regulation during anhydrobiosis^15^. Their possible role in anhydrobiotic animals was hypothesized by the discovery in the midge *P. vanderplanki* of the aquaporin PvAQP1^45^ that rapidly increase its expression at the onset of anhydrobiosis and it is thought to be required to remove the water from the body of the midge larvae^11,23,45^. Similarly, in the larvae of the goldenrod gall fly *Eurosta solidaginis,* the AQP3 protein which allows water and glycerol to cross the cell membrane is upregulated following desiccation, indicating that the larva is preparing itself for the osmotic stress associated with desiccation^83^. In tardigrades, the involvement of aquaporins during anhydrobiosis is supported by the expression of AQP4 in *M.* cf. *tardigradum*, at higher levels in the desiccated animals with respect to the active ones^84^. In the latter tardigrade species, eleven AQP transcripts (denoted MtAqp-1 through MtAqp-11) were identified^85^, with a high transcript abundance of MtAqp-3, -4, -5, -10. Our study provides evidence for the involvement of aquaporin 3 and aquaporin 10 during rehydration in tardigrades since the targeting for these two aquaporins in *P. spatialis* by RNAi has negative effects on animal motility immediately after rehydration process (t_0_). Since animals targeted for aquaporins are able to restore their motility in the course of the rehydration process (t_1_–t_48_), *P. spatialis* can desiccate even if the aquaporins are targeted. However, during rehydration tardigrades need more time to recover their motility, suggesting that aquaporins have a role during rehydration.

Although the molecular mechanisms underpinning anhydrobiosis are not yet completely understood, it seems clear that desiccation tolerance depends on the synergic action of an array of many different molecules working together. Our investigation supports this hypothesis and agrees with previous studies demonstrating that tardigrades species have a comprehensive number of genes encoding proteins involved in antioxidant defence^25^. Future studies on anhydrobiotic animals will further help in understanding the protectants and the mechanisms that contribute to their desiccation survival and could provide avenues for pursuing biotechnologies for the preservation, storage and distribution of human tissues and cells.

## Methods

### Collection of the target species

The eutardigrade *Paramacrobiotus spatialis* Guidetti et al. 2019 was used as target species. Specimens were collected from hazel-leaf litter at Formigine (80 m a.s.l.; 44° 34.253′ N, 10° 50.892′ E, Modena, Northern Italy). Leaf litter was immersed in tap water and then animals were extracted from their substrate by means of sieves, picked up with a glass pipette under a stereomicroscope and stored in tap water and natural mineral water 1:1 (rearing water) at 16 °C for 24 h before their use^10,31^.

### Experimental desiccation and rehydration of tardigrades

Animals were desiccated using a climate chamber (CHL, Angelantoni Industrie, Milan, Italy) that allows to control air temperature and relative humidity (RH) of the air. Animals were placed on a blot filter paper with a drop of rearing water and kept at 18 °C and 80% RH for 4 h, then to 50% RH for 4 h, and finally at 20 °C and 0–3% RH overnight^41^. At the end of these steps, the tardigrade body has the typical compact tun shape structure of a desiccated/anhydrobiotic tardigrade (Fig. 1d).

To rehydrate desiccated tardigrades, small amounts of rearing water were slowly added on each filter paper every 10 min for a total of 60 min, this rehydration step is here termed the “rehydration process”. Rehydrated tardigrades were maintained at 16 °C and observed under a stereomicroscope. Coordinated and active movements of the body (locomotion performance) constituted the criterion to evaluate tardigrade motility (survival). Locomotion performance was evaluated right after the rehydration process (t_0_) and after 1 h (t_1_) and 24 h (t_24_) (see Altiero et al.^34^).

### Detection of reactive oxygen species production under desiccation

The production of Reactive Oxygen Species (ROS) was detected after specimens of *P. spatialis* were kept in a desiccated state for 1 and 20 days (see above protocol) at 16 °C and 0–3% RH. In particular, ROS production was evaluated in coelomocytes (storage cells) 3 and 12 h after the rehydration process. As control, specimens kept in rearing water were used.

For each experimental conditions and controls, 2–5 animals were used, and for each animal 6–45 singular storage cells randomly chosen were analysed (Table S1).

The fluorescent probe 2,7-dichlorodihydrofluorescein diacetate (DCFH_2_-DA) diluted in methanol was used. It is the most common probe to detect overall level of intracellular ROS^55,56^. Preliminary tests evidenced that tardigrades survive when kept for 45 min in methanol used for dilution (methanol: distilled water, 1:10).

Each tardigrade was sonicated for 90 s in rearing water to allow the entrance of the probe in the tardigrade, and then incubated in 1 ml of 10 µM DCFH_2_-DA in methanol for 45 min at 20 °C in the dark. After these steps, each tardigrade was washed with rearing water, mounted on a slide with rearing water as mounting medium. Then, the animal was gently broken with a pressure on cover slip to obtain the release of isolated storage cells. Cells were observed using Leica TCS SP_2_ AOBS spectral confocal scanner microscope mounted on a Leica DM IRE_2_ inverted fluorescence microscope, with an Ar excitation laser (λ_exc_ = 488 nm) for DCFH_2_-DA (λ_em_ = 543 nm) available at Centro Interdipartimentale Grandi Strumenti (CIGS) of University of Modena and Reggio Emilia.

The ROS signal was measured quantifying the intensity of the fluorescence signal of each storage cell using the program ImageJ (National Institutes of Health). The fluorescence intensity of storage cells was standardised removing the storage cell size effect and the background signal.

The following formula was used:

Intensity of the storage cell signal = total fluorescence intensity of storage cell - [(x/y)·z].

In which: x = mean of two areas of the fluorescence intensity, y = mean of two background areas, z = storage cell area.

The mean value of fluorescence intensity of all previously measured storage cells was considered for each experimental condition (Table S2).

The statistical comparisons of ROS signals among the different experimental groups and controls were evaluated through the analysis of variance (one-way ANOVA) with a Tukey post-hoc test, using the program SPSS 23 (SPSS Inc., Chicago, IL. USA).

### Targeting of genes potentially involved in desiccation tolerance

The RNA interference technique was utilised to target eight tardigrade genes. The genes *gpx*, *gr*, *gst*, *cat*, *sod* and *tps* (encoding the enzymes glutathione peroxidase, glutathione reductase, glutathione transferase, catalase, superoxide dismutase, and trehalose-6-phosphate synthase, respectively), and *aqp 3* and *aqp 10* (encoding the membrane channel aquaporin proteins 3 and 10) were targeted in order to disrupt their function. To verify the involvement of these target genes in the desiccation tolerance of *P. spatialis,* three groups of animals were used: 1. uninjected control animals (control 1); 2. control animals injected with RNase free water (control 2); 3. injected animals with the dsRNA of a target gene (experimental group). For each target gene, three replicates each of 10 *P. spatialis* specimens (experimental group) were used. Each animal was injected with the dsRNA of a single target gene at the concentration of 4 µg/µl (see paragraph below). For both control groups, three replicates each of 10 animals were used.

The methodological steps applied to animals were: injection (except for control 1), desiccation, rehydration, and check of animal motility. The injection was performed following the protocol by Tenlen et al.^57^ with slight modifications. In particular, injection needles were pulled from borosilicate glass capillaries 1B100F-4 (Word Precision Instruments, *Inc*.) using a PC-10 Puller (Narishige). Injection slides were prepared as indicated by Tenlen et al.^57^. Injections of the animals were performed using an inverted microscope (Nikon Eclipse TE300) and a manual micromanipulator (Leitz Wetzlar) linked to a Hamilton Microliter™ syringe. Needle tips were broken by gently stroking the tips against the edge of a triangular coverslip piece to obtain an opening in the needle tip of 2–3 µm in diameter. The successful injection of dsRNA or RNAfree water into the body cavity of tardigrades was confirmed by the swelling of the body as indicated by Tenlen et al.^57^.

All injected tardigrades were transferred in rearing water and left overnight to allow the dsRNA to disrupt the gene function, as indicated by Boothby et al.^24^. Therefore, they were individually desiccated as described above, kept desiccated for 2 days, and rehydrated using the protocol described above. Tardigrade motility was recorded using the criterion of locomotion performance, which was evaluated right after the rehydration process (t_0_) and after 1 h (t_1_), 24 h (t_24_) and 48 h (t_48_).

The Kruskal–Wallis test was applied to compare the percentage of motile tardigrades (with locomotion performance) injected with dsRNA with respect to those of controls 1 and 2 at t_0_, t_1_, t_24_ and t_48_. The *tps* gene was chosen as a further control (Control 3) to exclude the possibility that the injection of large amount of dsRNA is harmful for *P. spatialis*, since animals targeted for this gene exhibited negligible effects on the motilities at every time of rehydration. The Kruskal–Wallis test was applied to compare the motility percentages of tardigrades injected with *tps* dsRNA with respect to those of animals injected with dsRNA of other target genes at t_0_, t_1_, t_24_ and t_48_. The statistical analysis was performed with the program SPSS 23 (SPSS Inc., Chicago, IL, USA).

### Synthesis of specific target gene dsRNA

The transcriptome of *P. spatialis*^24,86^ was compared with the transcriptome of the eutardigrade *Hypsibius exemplaris* Gąsiorek, Stec, Morek & Michalczyk, 2018^24^ and with sequences of the nematode *C. elegans* Maupas, 1900^87^ contained in WormBase and GenBank. Obtained sequences were aligned using the program BLAST to design gene-specific primers (Table S4). Genomic DNA was extracted from five specimens of *P. spatialis* using the Worm Lysis Buffer^57^. Gene-specific primers (Table S4) were used to amplify the target sequence with these cycles: first denaturation step for 2 min at 95 °C, at least 15 cycles with 30 s at 95 °C, annealing at 70 °C for 1 min with a decreasing rate of 1 °C/cycle until the melting temperature (T_m_; Table S4) of the tested primers is reached, 2 min at 72 °C, 30 cycles with 30 s at 95 °C, annealing at T_m_ of the tested primers for 1 min, and 2 min at 72 °C, with a final elongation step at 72 °C for 5 min. PCR products of each target gene were directly inserted into a plasmid vector (pCR™ -Blunt II-TOPO® vector) and cloned using the Zero Blunt TOPO PCR Cloning Kit (Thermo Fisher). The recombinant vector was then used to transform competent Mach1™ T1^R^ cells of *Escherichia coli* (Thermo Fisher). To verify if the target gene has been inserted in the vector, a PCR reaction was performed using the purified plasmid DNA as a template and gene-specific primers, with this cycle: 30 cycles with denaturation at 95 °C for 30 s, annealing at 53 °C for 30 s min, and elongation at 72 °C for 1 min, with a final elongation step at 72 °C for 5 min. After successful control of the amplification, several clones were sequenced for each gene, using gene-specific primers. After sequencing, the plasmid sequence was aligned with the target gene sequence using the program BLAST to check the real insertion of the target gene in the plasmid and the absence of introns in the sequence. New primers were designed by adding the sequence of promoter T7 (5′-TAA TAC GAC TCA CTA TAG GG-3′) to the 5′ portion of each original gene specific primer sequence (Table S4) and they were used to synthesize dsRNA from the *P. spatialis* genomic DNA, using the T7 RiboMAX™ Express RNAi System (Promega), following the manufacturer’s protocol. Following ethanol precipitation, dsRNA of each target gene was resuspended in nuclease-free water and was ready to be used in RNAi. The targeted genes and their sequence references are reported in Table S2. The list of targeted sequences is reported in the supplementary section.

### Protocol to verify the successful targeting of genes by RNAi

A protocol was designed to verify if RNAi led to a decrease of each target gene expression level. A new group of five specimens of *P. spatialis* were injected with each target gene specific dsRNA, then individually desiccated, and rehydrated following the protocols described above. At t_0_ (after the rehydration process), total RNA was extracted from each animal using the MasterPure Kit (Epicentre), and then used to synthesise cDNA using the SuperScript® III First-Strand Synthesis System for RT-PCR (Thermo Fisher), following the manufacturer’s protocol. Then, each target gene was amplified using the specific primers designed on *P. spatialis* transcriptome (Table S4) and applying the same thermal cycling conditions described above. As a PCR control, the same RNA extracted from each individual tardigrade was used to amplify the DNA polymerase II, which was used as control gene (accession number: GFGY01000030; position: 7492–7959; length: 468 bp; targeted sequence in supplementary section). For this gene, specific designed primers (F- 5′-GTC ACG GAC GAA GGA GAA TTT A-3′; R- 5′-ACG TGA AGA TGG GCG TAT TG-3′) were used.

## Supplementary Information

Supplementary Information.
