# Supplementary material for: Production of reactive oxygen species and involvement of bioprotectants during anhydrobiosis in the tardigrade Paramacrobiotus spatialis
Source: Sci Rep. 2022 Feb 4;12:1938. doi: 10.1038/s41598-022-05734-6 (PMC8816950; doi:10.1038/s41598-022-05734-6)
Supplement: Supplementary file 1 — Supplementary Information. [file 41598_2022_5734_MOESM1_ESM.docx]

**Production of reactive oxygen species and involvement of bioprotectants during anhydrobiosis in the tardigrade *Paramacrobiotus spatialis*.**

Ilaria Giovannini^1*^, Thomas C. Boothby^2-3^, Michele Cesari^1^, Bob Goldstein^3^, Roberto Guidetti^1^, Lorena Rebecchi^1^

^1^Department of Life Sciences, University of Modena and Reggio Emilia, Modena, Italy

^2^Department of Molecular Biology, University of Wyoming, Laramie, Wyoming, USA

^3^Department of Biology, University of North Carolina at Chapel Hill, North Carolina, USA

**SUPPLEMENTARY SECTION**

**Table S1.** Number of animals and storage cells for each specimen of *Paramacrobiotus spatialis* used to detect the intracellular ROS production in control and treated specimens.

| **Experimental group** | **Animal** | **Number of storage cells** |
| --- | --- | --- |
| **Control** | 1 | 28 |
|  | 2 | 32 |
|  | 3 | 40 |
|  | 4 | 23 |
|  | 5 | 15 |
| *Total number of cells* |  | **138** |
| **D 1 R 3** | 1 | 21 |
|  | 2 | 25 |
| *Total number of cells* |  | **46** |
| **D 1 R 12** | 1 | 15 |
|  | 2 | 18 |
|  | 3 | 45 |
| *Total number of cells* |  | **78** |
| **D 20 R 3** | 1 | 20 |
|  | 2 | 6 |
|  | 3 | 32 |
|  | 4 | 20 |
| *Total number of cells* |  | **78** |
| **D 20 R 12** | 1 | 15 |
|  | 2 | 14 |
|  | 3 | 7 |
| *Total number of cells* |  | **36** |

D 1 = animals kept in a desiccated state for 1 day, D 20 = animals kept in a desiccated state for 20 days, R 3 = 3 h after the rehydration process, R 12 = 12 h after the rehydration process.

**Table S2.** List of the targeted sequences of genes, their GenBank accession number and location in the transcriptome of *Paramacrobiotus spatialis*^24, 86^.

| **GENES** | **ACCESSION NUMBER** | **POSITION** | **LENGTH (bp)** |
| --- | --- | --- | --- |
| Catalase (*cat*) | GFGY01006088 | 828-1363 | 536 |
|  |  |  |  |
| Glutathione peroxidase (*gpx*) | GFGY01022429 | 713-909 | 197 |
|  |  |  |  |
| Glutathione reductase (*gr*) | GFGY01009280 | 381-708 | 328 |
|  |  |  |  |
| Glutathione transferase (*gst*) | GFGY01012310 | 1364-1522 | 159 |
|  |  |  |  |
| Superoxide dismutase (*sod*) | GFGY01007093 | 622-1057 | 436 |
|  |  |  |  |
| Aquaporin 3 (*aqp 3*) | GFGY01018572 | 816-1219 | 404 |
|  |  |  |  |
| Aquaporin 10 (*aqp 10*) | GFGY01008576 | 1457-1870 | 414 |
|  |  |  |  |
| Trehalose-6-phosphate synthase (*tps*) | GFGY01003581 | 2362-2980 | 619 |

**Table S3.** Statistical comparisons (Kruskal-Wallis test) between motility percentages at different times after rehydration (t_0_, t_1_, t_24_ and t_48_) of target genes with respect of the motility percentages at the same time after rehydration of *tps* gene (non-effective gene) in *Paramacrobiotus spatialis*. Number of pairwise comparisons = 132; n.s. = not significant.

|  | | ***gpx*** | ***gr*** | ***gst*** | ***cat*** | ***sod*** | ***aqp3*** | ***aqp9*** |
| --- | --- | --- | --- | --- | --- | --- | --- | --- |
|  |  | **t_0_** | | | | | | |
| ***tps* (non-effective gene)** | **t_0_** | *p* < 0.01 | *p* < 0.05 | n. s. | *p* < 0.05 | n. s. | *p* < 0.05 | *p* < 0.05 |
|  |  | **t_1_** | | | | | | |
|  | **t_1_** | *p* < 0.01 | n. s. | n. s. | n. s. | n. s. | n. s. | n. s. |
|  |  | **t_24_** | | | | | | |
|  | **t_24_** | *p* < 0.05 | n. s. | n. s. | n. s. | n. s. | n. s. | n. s. |
|  |  | **t_48_** | | | | | | |
|  | **t_48_** | *p* < 0.05 | n. s. | n. s. | n. s. | n. s. | n. s. | n. s. |

**Table S4.** List of the specific primers designed for each target genes of *Paramacrobiotus spatialis*.

| **GENES** | **PRIMERS**  **Forward (F) and Reverse (R)** | **MELTING**  **TEMPERATURE (T_m_)** |
| --- | --- | --- |
| Catalase (*cat*) | F- 5’-GGA GAC GAT GCA CAC CAT TAT-3’ | 55 °C |
|  | R- 5’-GGG ACA AAC TGG ACG ATT GA-3’ |  |
| Glutathione peroxidase (*gpx*) | F- 5’-AGT TCC TCC GTA CTT CCC T-3’ | 53 °C |
|  | R- 5’-TCA CGA TGA CCA GCA CTT TAC-3’ |  |
| Glutathione reductase (*gr*) | F- 5’-AAA CGG AGC AGT GAC CAA AC-3’ | 53 °C |
|  | R- 5’-TAA ATC GTC CCA CAC CGG TAAC-3’ |  |
| Glutathione transferase (*gst*) | F- 5’-GGC GCA CAA GGA TTC TCA TTA-3’ | 53 °C |
|  | R- 5’-CGA GGA TTT GCC CAT CAA CT-3’ |  |
| Superoxide dismutase (*sod*) | F- 5’-TGT GTC TGC GTT CTG GTA TG-3’ | 53 °C |
|  | R- 5’-CGT TAT CGC AAC CGT CTC TAA-3’ |  |
| Aquaporin 3 (*aqp 3*) | F- 5’-GGC TGA ATT CTT CGG GAC AA-3’ | 50 °C |
|  | R- 5’-CGG TGA ATC GAC TGA AGG TAT C-3’ |  |
| Aquaporin 10 (*aqp 10*) | F- 5’-CGA TCT TGG TCC GCG TAT TT-3’ | 53 °C |
|  | R- 5’-ACG TGA AGA TGG GCG TAT TG-3’ |  |
| Trehalose-6-phosphate synthase (*tps*) | F- 5’-TCC GTT GAT GTG GTC GAT ATG-3’ | 50 °C |
|  | R- 5’-CTG GGT GAG AAA CAC TGG ATA G-3’ |  |
|  |  |  |
|  |  |  |

**Figure S1.** Relative expression of *DNA pol II* and target genes amplified by RT-PCR in *Paramacrobiotus spatialis*. **(a)** Non-injected control animal; 1: DNA polymerase II (*DNA pol II*), 2: glutathione peroxidase (*gpx*), 3: glutathione reductase (*gr*), 4: glutathione transferase (*gst*), 5: catalase (*cat*), 6: superoxide dismutase (*sod*), 7: trehalose phosphate synthase (*tps*), 8: aquaporin 3 (*aqp 3*), 9: aquaporin 10 (*aqp10*). **(b-i)** Animals injected with dsRNA of target gene; 1: *DNA pol II*, 2: *gpx*, 3: *gr*, 4: *gst*, 5: *cat*, 6: *sod*, 7: *tps*, 8: *aqp 3*, 9: *aqp10*. The molecular weight marker (GeneRuler 100 pb Plus DNA Ladder, Thermo Fisher) was loaded in the first (a-i) and last well (a). Full-length gels are presented in Supplementary Figure S2.

**
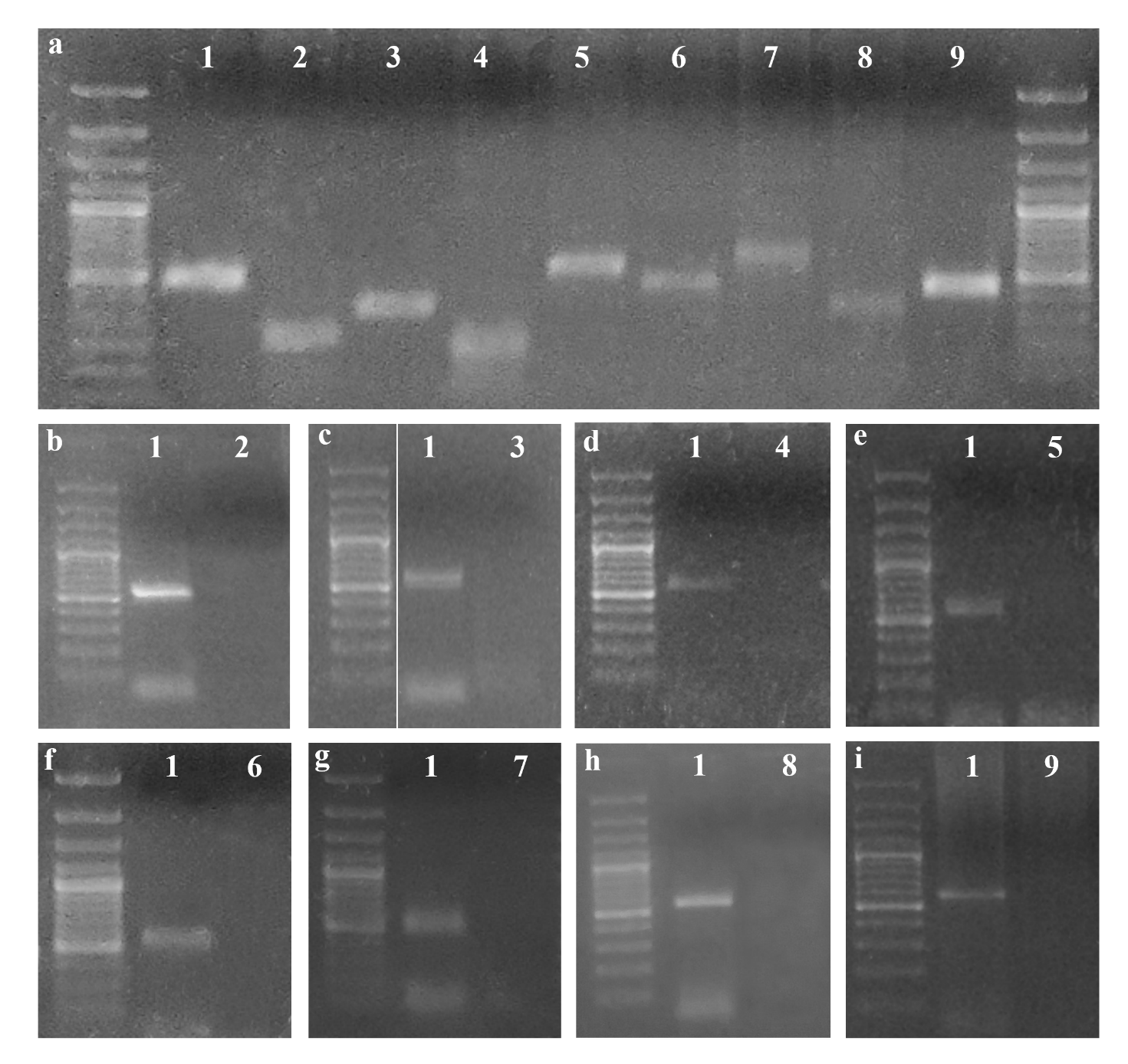
**

**Figure S2.** Original images used to create the Figure S1 about the relative expression of *DNA pol II* and target genes amplified by RT-PCR in *Paramacrobiotus spatialis*. Black boxes outline the edges of the blots. Red boxes denote the regions of the original blots used to create the Figure S1. The letters in red boxes match the original images to the cropped version of images reported in Figure S1. The unused sets of bands are: (b,c,d,h) un-injected control animals, (e) injected animal where the samples were unfortunately loaded in the inverted order in the gel, i.e. no amplified band of catalase in the third well and the amplified band of DNApol in the fourth well; (i) injected animal.


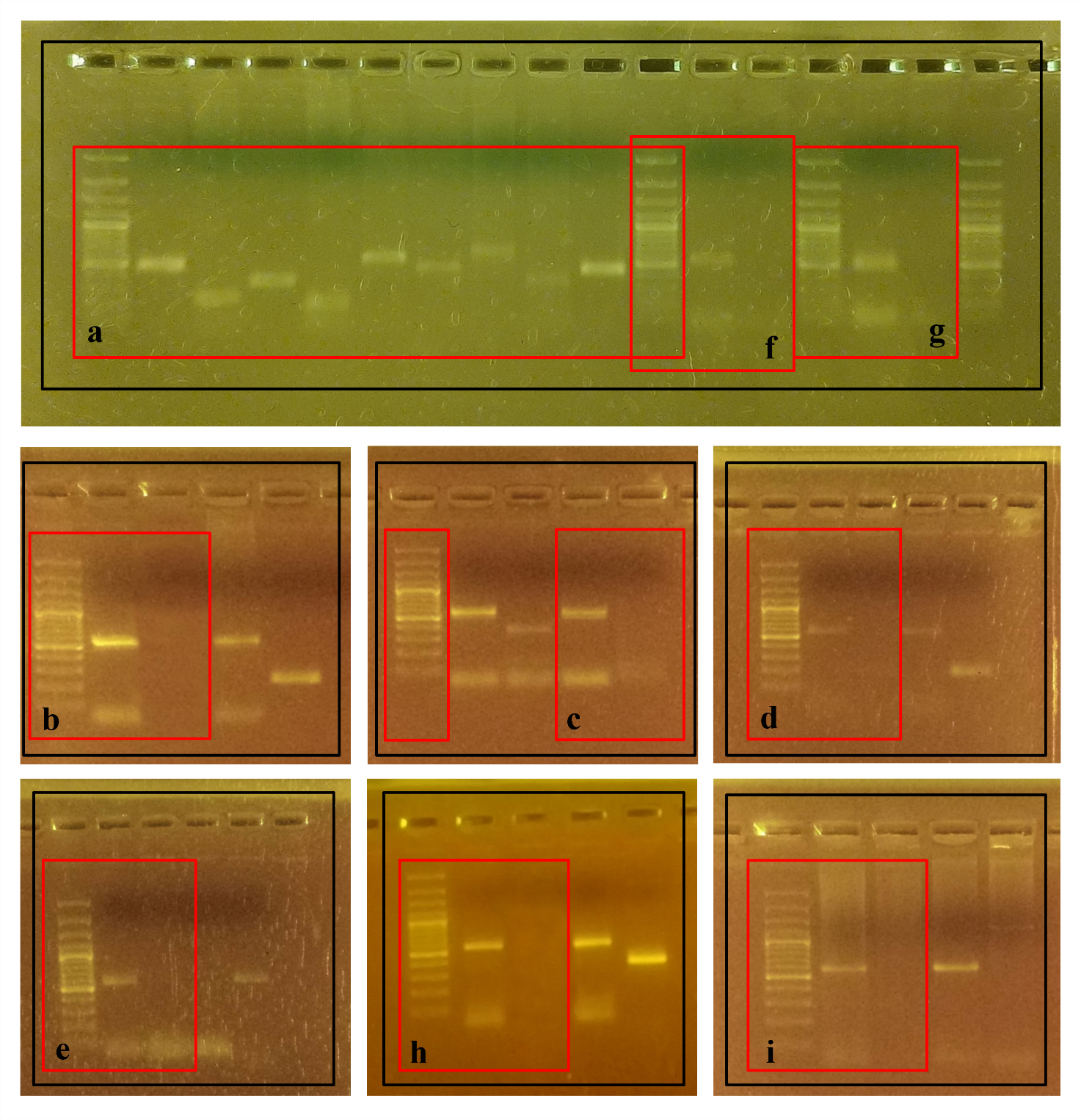


**List of targeted sequences**

***cat***

GTGGTTGATGTCTGATCGGGCCATTCCCCGTTCATACTCAATGATGGAGGGTTTCGGTGTCCACACTTTCCGTCTGGTCAACGAGGAGGGCGTTTCCCGTTTCGTGAAATTCCATTGGAAACCCGTCCTGGGAGTCCACTCGTTGGTGTGGGACGAAGCCACAAAGATCAGTGGTGCCGATCTGGATTTCAACCGTCGACAGCTGTGGGAGAGCATTGAAGCTGGAAATTATCCTGAATGGGAATTGGGACTTCAGGTTGTGGAGGAAGCGGATGAATTTAAATTCGACTTTGATCTGTTGGATCCGACGAAAATCATCCCGGAGGAGTTGGTGCCAGTGCAGCGCATTGGGCGCCTGGTGCTGAATCGTAATCCGGACAACTTTTTCTGTGAGACGGAACAAGTCGCGTTCTGCGTCTCGCACATTGTGCCCGGCATTGACTTCAGCAACGATTTGTTGATGCAAGGTCGTTTGTTTTCGTATTTGGATACGCAATTGAGCCGTCTGGGTGGCGCAAACTTTCACGAGATTCCCA

***gpx***

GTCCTGCTTCTGTGGTAGAGGTGGAATTCCGTATGAAATCTCCTTACGGTTACTGGCTGGGCTGCGTTGAAGGGTTCTATGGCTGTATGGGTTACGGTCAAGCACGGCATATTTAAAAAAGTTTTACTGTTACGGTTTCGCATTGCTTTTTAAGGCTACGGTAGGGTACGGCACATCTATGGCACTTTATTACTTAT

***gr***

GCAGTATGATTTGGTTGTGCTTGGCGGAGGATCAGGTGGTATCGCGACGGCCAGAAAAGCAGCCAGCTTGGGCGCTAAAGTTGCTCTTATTGAGTCGCGGGAAGGGCGTCTTGGAGGAACTTGTGTTAATGTCGGTTGTGTACCAAAAAAAATCACATGGAATGCCTCCAGTCTAGCCGCGAGTATTGAACATGATTTAAAGGATTACGGTTTTGACATCGAATACAAGGGTTTTGACTGGAAGAAGTTTAAAGCCAAACGGGATGCATACGTTAGAAAACTAAACGGAATATATGACAATAATTTGAAAAAGGATGGAATTGAACTG

***gst***

TAAATTAACGTACTTTAATTTACGAGGCATTGCTGAGCCCATTCGGCAGGTCTTTGCGTACGCAAATGTGCCATATGAGGATGTACGCCTTGAGCGGGATCAGTGGGCTGATTCGTCCCAAAAACAGCAAGCACCGTTTCACCAGATGCCAATCCTGGA

***sod***

GGGCAATGTCACCGCTGGCTCGGACGGTACTGTGAAACTGAGCATCGAGGATTCTCAGATTTCTCTGGAAGGGCAGAACAACATTGTTGGCCGTGCCATGGTGGTGCACGAGAAGAAGGACGATTTGGGCAAGGGCGGTGATGACGAGAGCCTCAAGACCGGTAACGCTGGACCCCGTCTGGCTTGCGGAGTGATTGGAGTCACCAAAAATCAATAGTTTTTTGTTCATTAGCTTGAGAATCACAACCGTCTGATTTCGTAAGTTACTTTAATGGCTACATTCGCAGTTTTTAGGTTGCTTTACTTTGATACACGTGTATCTTTTATAACTTAATATGTCGGTGGAGGTTAACAAAATGTTCGTTTGGTGCTTGGTTGGGGTATGCGAACCCGGTTAATTTTTGAAGACGGTCAGTAAGAAGTGCATACATTAAAT

***aqp3***

ATGACGTAGATGCCGGCCGAGGCCACCAAGGCGCCCAAGTACTGCGCGGCCATGTAGATGAACATTTTCTTCCACGAGATCCGCCCCATCACGGCGAAGGCCAACGATACCGCCGGGTTGATGTGGGCGCCGGAAACGCCGCCGCACACCAACACCCCCATCATCACGGCCAGTCCCGCGCCGAGAATCAGGTGGACATGATCCGCACCCGTGGTTAACATGTAGTGTCCATCGGGTGACTGTTTCATCCGGTTAAACGTCGCATGCGCCGCCATCGCGTCGATCATGTACACAAGGACCATGGTTCCAAAGAATTCAGCCAGAAAACACCGAACGTAATCATGTTGAAAGCTCACAGCGCGTTTCACGGTCCGACGCCAGGAGACGCTCATTTTTCACAATCC

***aqp10***

TTACAGCCATGGCGGGATGGGGAGTGGAAACGTTTAGCTTCCGAAATCACTCTTGGTTTTGGGTGCCGATCATCGGTCCGCATCTGGGAGCGCTCTTGGGGCTAGGAATTTACCAGCTGCTTATCGGGAATCAGTGGCCAGAACTGAAACGTACAGGAATTGAACTACAGGTTGTACGGAAAGCCGACGAATACGAGCTGGTCAAAGCGAGCCACGTGACACCCCATGACGCCACCGTTCCCGTATCCTTCCGTACAGACGACGAACAAACTCAACGTCTTTTGCATGTCCGTTAAACAAAGCATTCAGTTAGAAAAAGGGAATGGAATTGCAGATCTTAACACATGCGGCATCTCCGAAATTCTCCATTGGTGCATTAACTCCCGTCGCAAAGTACTCAAATCTTGAGTCGAC

***tps***

ATGGAATTTTTGAGCGTTTACCGGGAAAGAAGAAATGGCGCAGTTTCCGCAGTGCCGACTGGCAGCCGAAAATCTTGGTGCATCTGCAAAAGTTTTCCGAATTAACACCCGGAAGTGAGATTGAGACCAAGGATTGCTCGCTGGTGTGGCATTATGATAACGCCTCGGCATACGATGCACAGAAACACCTCCTGGAACTGAAACGGGTTATCAGTCCGTTGGCTAATGTGTGGAAATTGGATCTGATCCAGGGGAATATGACACTGGAGATCCGGCCGAAAGGACGCAGCAAGGGCAGTACGGCGAAAGAACTGGTCGGACGGAATTCGTATGATTTTGTCCTGGTGGTGGGGGATGATCAGGATGACGAGAGTATTTTCACTGACTGGCCGGATGAGGCTTACACGGTTAAAGTCGGTCATGGGCGGACCGGCGCCCGGCTGCGCGTTTTCCGGCCGGAAGAGGTGGTGCATCTGCTGAGAAAGTTTATCAGTAGCGGCAGTATATTTGCTGTTTCGGCGTAGTTGTAGATCTCTTGTTTCTTTCTCTCTTCGGGATGTGGTCCGAGTGTGCTCTGCGATAGTGGTGCGTTTGGTCCTTGTCAATATGGTTCGATTTA

***DNA pol II***

AAGCCATATCGGAATGGATTTTGGAAACGGATGGCACAAGTCTTATGCGGGTTCTTTCCGATCGCGATGTCGATCCTGTGCGAACTGTCAGCAACGACATTTGCGAAGTGTTTGCGGTTCTTGGCATTGAAGCGGTTCGAAAATCGCTGGAAAAAGAAATCAATCACGTCATTTCGTTCGACGGTTCATACGTTAACTACCGTCATCTCGCGCTTCTGTGTGATGTGATGACAGCGAAAGGCCATCTTATGGCTATCACGCGCCACGGTATCAACCGGCAAGAAGTTGGACCTCTGATGAAATGCTCATTCGAAGAGACAGTTGATATTCTGTTGGAAGCAGCGGCCCACGCTGAATGCGATTATTTGAAGGGTGTCTCTGAGAATATTATGCTGGGTCAGTTGGCACGTTTGGGCACGGGTGCTTTCGATCTCATCTTGGACCCCGACAAGTGCCGATATGGTATGG
